# Usefulness of Organic Acid Produced by *Exiguobacterium sp.* 12/1 on Neutralization of Alkaline Wastewater

**DOI:** 10.1100/2012/345101

**Published:** 2012-04-30

**Authors:** Niha Mohan Kulshreshtha, Anil Kumar, Gopal Bisht, Santosh Pasha, Rita Kumar

**Affiliations:** ^1^Environmental Biotechnology Division, Institute of Genomics and Integrative Biology, Mall Road, Delhi 110007, India; ^2^Department of Biotechnology, University of Pune, Ganeshkhind, Pune 411007, India; ^3^Patent Division, National Institute of Immunology, Aruna Asaf Ali Marg, New Delhi 110067, India; ^4^Peptide Chemistry Division, Institute of Genomics and Integrative Biology, Mall Road, Delhi 110007, India

## Abstract

The aim of this study was to investigate the role of organic acids produced by *Exiguobacterium sp.* strain 12/1 (DSM 21148) in neutralization of alkaline wastewater emanated from beverage industry. This bacterium is known to be able to grow in medium of pH as high as pH 12.0 and to neutralize alkaline industrial wastewater from pH 12.0 to pH 7.5. The initial investigation on the type of functional groups present in medium, carried out using FT-IR spectroscopy, revealed the presence of peaks corresponding to carbonyl group and hydroxyl group, suggesting the release of carboxylic acid or related metabolic product(s). The identification of specific carboxylic group, carried out using RP-HPLC, revealed the presence of a single peak in the culture supernatant with retention time most similar to formic acid. The concentration of acid produced on different carbon sources was studied as a function of time. Although acid was present in same final concentration, the rate of acid production was highest in case of medium supplemented with sucrose followed by fructose and glucose. The knowledge of metabolic products of the bacterium can be considered as a first step towards realization of its potential for large-scale bioremediation of alkaline wastewater from beverage industry.

## 1. Introduction

Alkaliphiles—microorganisms that have pH optima for growth at or above pH 9—have made a great impact in industrial applications. Biological detergents contain enzymes, such as alkaline cellulases and/or alkaline proteases, that have been produced from alkaliphiles [1, 2]. Alkaliphiles have also been utilized for the industrial production of enzymes for that could be of specific use, for example, cyclodextrin by alkaline cyclomaltodextrin glucanotransferase [1] and alkaline active maltohexaose-forming *α*-amylase [3] which find application in foodstuff, chemical, and pharmaceutical industries. It has been reported that alkali-treated wood pulp could be biologically bleached by xylanases produced by alkaliphiles [4]. Fujiwara and coworkers [5, 6] have reported the use of an alkaline protease to decompose the gelatinous coating of X-ray films, from which silver was recovered. Alkaliphiles have also proved their potential in biodegradation of a variety of organic compounds [7–10].

Thus, alkaliphilic bacteria have attracted much interest because of their extracellular enzymes and biochemical properties such as alkaliphily and alkali stability. Their bioenergetics has also been investigated in some detail [11], whereas little is known about their physiology, for example, intracellular enzymes and metabolites. Features of the intermediate metabolic process are important since they aid in characterizing the bacterium, its enzyme composition, the metabolic stage of cells, and the possibilities for metabolic engineering. The ability of alkaliphiles to strongly fluctuate the pH of carbohydrate containing medium [12] was exploited in previous work for neutralization of highly alkaline wastewater emanated from beverage industry using *Exiguobacterium sp.* strain 12/1 [13]. Genus *Exiguobacterium *belongs to the order Bacillales which also includes members of genus *Bacillus*. *Exiguobacterium sp.* 12/1 is a facultative alkaliphile which grows optimally at pH 10 and is capable of neutralizing alkaline wastewater to bring it down from pH 12.0 to pH 7.5. It is assumed that the bacterium releases some acidic metabolic product(s) in order to neutralize the highly alkaline external medium. However, it is important to characterize the type of metabolites released into the extracellular medium. Here, we study the production of organic acids as a possible mechanism for alkali neutralization. Such types of studies will be necessary before the large-scale applications of the bacterium for neutralization of alkaline wastewater from beverage industry could be developed.

The major carbon source in soft drink industry wastewater is sucrose (disaccharide containing glucose and fructose) which is also the major contributor to its biochemical oxygen demand (BOD) [14]. The average BOD of soft drink industry wastewater ranges from 600 to 4500 mg/L which is equivalent to 673–5052 ppm sucrose. A literature survey of the metabolic products of a large number of bacteria growing on simple sugars suggests that the bacteria could be utilizing these simple sugars to generate organic acids. This is further corroborated by the analysis of extracellular metabolic products of other alkaliphilic Bacillus species [15]. The major organic acid produced on sucrose carbon source in these studies was found to be acetic acid. Formic acid is a common metabolite of neutrophilic bacteria in anaerobic conditions, while *B. circulans* var. *alkalophilus* produces up to 2 g/L of it even in aerobic cultures. Other volatile acids such as propionic, butyric, isobutyric, and isovaleric acids are typical for strains *Bacillus alcalophilus ssp*. *halodurans*, *B. alcalophilus,* and *Bacillus sp.* 17-1. Isobutyric and isovaleric acids have been reported in the media of several neutrophilic bacilli [16, 17]. But these acids, as well as propionic and butyric acids, are considered to originate from amino acids based on studies on *Clostridium sp*. [18]. Lactic and pyruvic acids are quite commonly produced by neutrophilic bacilli [16, 19, 20], but the production of succinic acid by *Bacillus* is rare [21]. Ethanol has not been detected in alkaliphilic bacilli [15] even though it is a typical product of glucose cultures of many neutrophilic bacilli [21, 22]. Thus alkaline growth conditions may affect the production of the neutral metabolites [15]. In this study we have used reverse phase high-performance liquid chromatography to study type and concentration of acids produced by *Exiguobacterium sp.* strain 12/1 during neutralization of medium of high pH containing different types of carbon sources.

## 2. Materials and Methods

### 2.1. Strain and Culture Conditions

The *Exiguobacterium sp*. 12/1 culture was obtained from DSMZ (DSM 21148) and was maintained as glycerol stocks. Alkaline basal medium (ABM) containing (all concentrations in g/L): peptone, 1; yeast extract, 0.5; glucose, 1; K_2_HPO_4_, 0.1; Na_2_CO_3_, 1; pH 10 (the last three components added to the autoclaved medium from separately sterilized solutions) was used for routine cultivation of strain 12/1 at 37°C. For IR and RP-HPLC analysis, the bacterium was grown at 37°C, 200 rpm in minimal salt medium (MSM) containing (all concentrations in mM): K_2_HPO_4_, 10; KH_2_PO_4_, 10; MgSO_4_·7H_2_O, 1; EDTA disodium salt, 0.3; ZnSO_4_·7H_2_O, 0.01; MnSO_4_, 0.02; CuSO_4_·5H_2_O, 0.004; FeSO_4_·7H_2_O, 0.1; NaMoO_4_·2H_2_O, 0.004; (NH_4_)_2_SO_4_, 5 and 1% (w/v) of one of the following carbohydrates: glucose, fructose, or sucrose (all the components added from the separately autoclaved concentrated stock solutions). Final pH of the medium was adjusted to 10.5 using 1 N NaOH.

### 2.2. Analysis of Growth and Culture pH

1 mL of log phase culture on ABM was used to inoculate precultures (50 mL) (MSM containing 1% sugar). The actual test culture (250 mL MSM in 500 mL Erlenmeyer flask) was inoculated with entire preculture in mid-log phase (O.D. ~ 1.2). Each culture set consisted of three flasks. The absorbance of samples at 650 nm was used as the measure of bacterial growth. The pH was determined in cell-free culture samples at room temperature after centrifugation 4000 ×g for 20 min.

### 2.3. FT-IR Analysis

Culture was harvested after 60 h of growth and was centrifuged at 4000 ×g for 20 min. For IR analysis, the culture supernatant was freeze dried and crushed into powdered form. The powdered supernatant was then mixed with potassium bromide, and the mixture was pressed into tablet. Finally, the tablet was analyzed by using the FT/IR-4200 spectrometer (JASCO, Tokyo, Japan).

### 2.4. RP-HPLC Analysis

Culture was harvested at different time points and was centrifuged at 4000 ×g for 20 min. For HPLC analysis the culture supernatant was filtered through 0.22 *μ*m filter, and 10 *μ*L of filtered sample was injected into the HPLC column.

Analytical standard-grade formic acid, acetic acid, succinic acid, propionic acid, lactic acid, and isobutyric acid were obtained from Sigma. Stock standard solutions (100 mg/mL or 100 *μ*L/mL) were prepared and were stored at 4°C for further use. Working standard solutions (10 mg/mL or 10 *μ*L/mL) were prepared daily. Milli-Q water (Millipore) was used to prepare buffer and stock solutions of each compound and samples. The stock solutions, samples, and buffer were filtered through cellulose membrane filters Whatman (0.45 *μ*m, Whatman, Clifton, NJ, USA). The solvents were degassed under vacuum prior to use.

The organic acid analysis was done according to the method of Tormo and Izco [23]. The analysis was carried out on a Breeze System (Waters, Mildford, MA, USA) consisting of a 1525 binary HPLC pump, a 717 plus autosampler, and a 2487 two channel UV detector set at 210 nm, operated using a Breeze software. The separation was performed on an Atlantis dC18 column (Waters) 250 × 4.6 × 5 *μ*m. 20 mM of NaH_2_PO_4_ adjusted to pH 2.20 with phosphoric acid was prepared daily and filtered through 0.2 *μ*m hydrophilic membranes (Millipore). The solvent programme utilized two reservoirs containing 1% of acetonitrile in 20 mM phosphate buffer adjusted to pH 2.20 with phosphoric acid (Solvent A) and acetonitrile (Solvent B); the flow rate was set at 1.5 mL/min at room temperature. The gradient programme started with 100% of solvent A and after 7 min Solvent B was increased linearly to reach 7% in 5 min. From 12 to 19 min the rate was kept at 93% of Solvent A and 7% of Solvent B. After that the rate was changed to the starting conditions to equilibrate the column for 15 min before injecting again 10 *μ*L of the next sample.

## 3. Results

### 3.1. Analysis of Neutralization on Defined Medium

**Figure 1:**
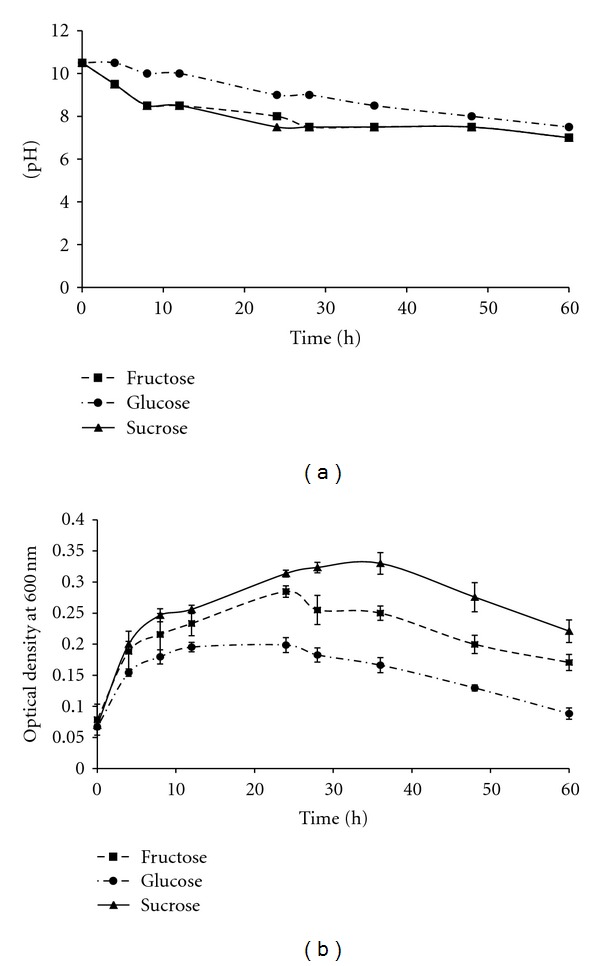
Variation in pH (a) and O.D. (b) with time on MSM. The values represent average of three replicate measurements, and error bars represent standard deviation.

 Minimal salt medium was selected for the analysis of organic acid produced by the bacterium because of its defined nature and similar carbon source as beverage industry wastewater. The bacterium was allowed to grow in minimal salt medium supplemented with different carbon sources glucose, fructose, and sucrose.  Figure 1 shows the growth profile and pH characteristics of the medium with time. Fructose and sucrose yielded a much faster neutralization of the medium as compared to glucose. The final pH obtained with glucose was also slightly higher than that obtained in case of sucrose- and fructose-supplemented medium. This is also reflected in the growth profile of the bacterium grown on the three carbon sources. The bacteria grew more rapidly in sucrose followed by fructose and glucose.

### 3.2. Identification of Functional Group Present in the Culture Supernatant

To identify the functional broad functional group of the metabolite produced by the bacterium in order to neutralize alkaline wastewater, the freeze-dried culture supernatant was subjected to FT-IR spectroscopy. Two peaks corresponding to carbonyl group (at 1644.98 cm^−1^) and hydroxyl group (at 3436.74 cm^−1^) were present in the spectrum (Table 1). According to the literature survey, the bacterium most likely produces organic acids as a metabolic product which neutralizes the alkaline wastewater.

### 3.3. Identification of the Specific Metabolic Product of the Bacterium

**Figure 2:**
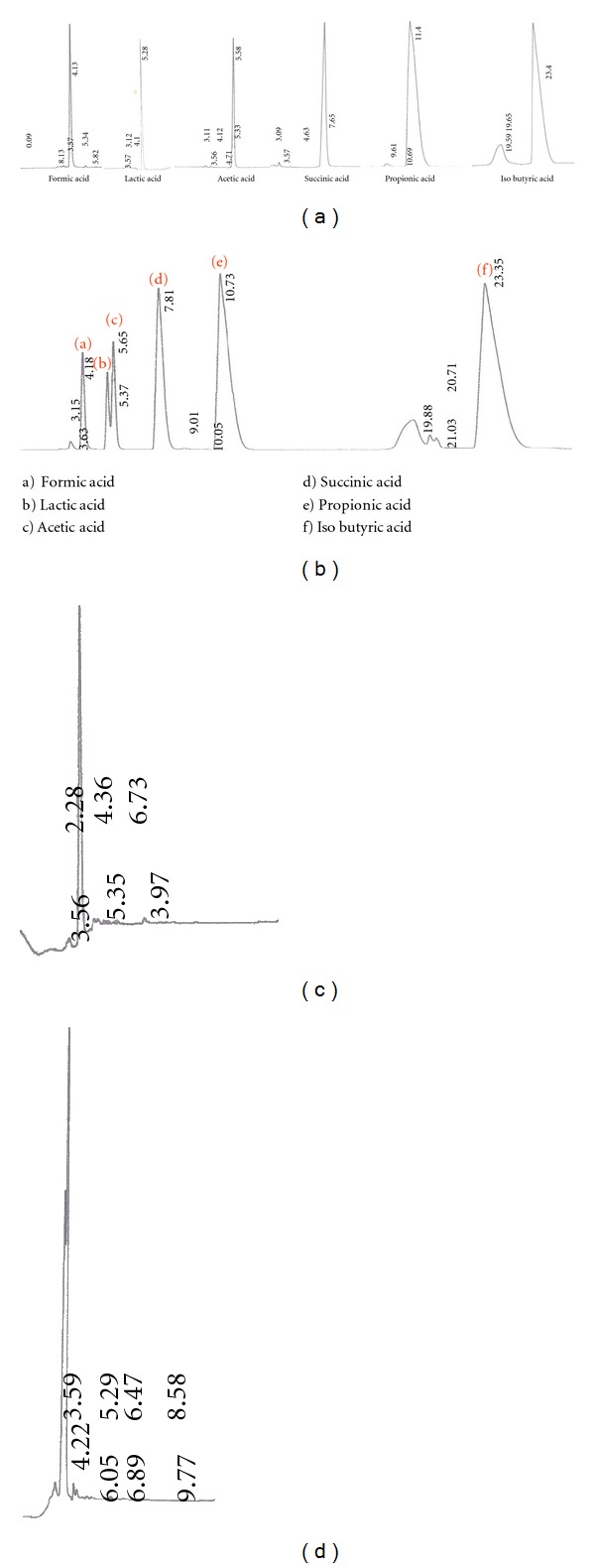
RP-HPLC chromatograms of individual standard organic acids (a), standard organic acids in mixture (b), culture supernatant (c), and culture supernatant spiked with formic acid.

In order to identify the organic acid produced by the bacterium, Reverse Phase HPLC was performed using known organic acid standards selected after literature survey. The standards were run both individually (Figure 2(a)) and in mixture (Figure 2(b)) in order to ascertain any differences in retention time arising due to interference by other organic acids in the medium. The RT of standard organic acids in the two cases was found to be similar with the difference in retention time not exceeding 0.09 units except for propionic acid (Table 2). The culture supernatant was analyzed by the same method and was found to comprise of a single peak with retention time similar to formic acid. This was further confirmed by spiking the supernatant with standard formic acid whose peak superimposed with that of the product in supernatant (Figure 2(d)).

### 3.4. Quantitative Analysis of the Metabolic Product of the Bacterium

**Figure 3:**
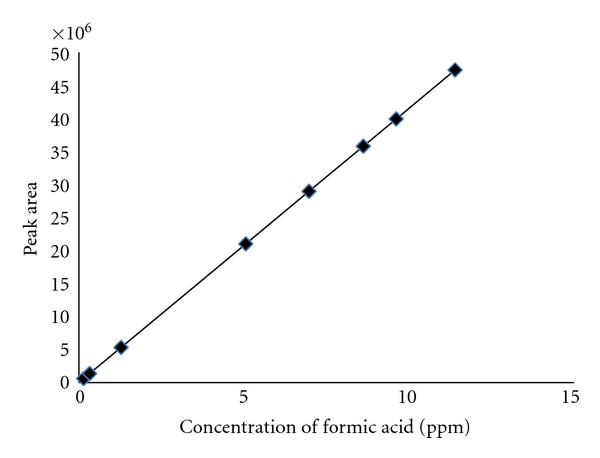
Standard curve for the determination of concentration of formic acid in the culture supernatant.

**Figure 4:**
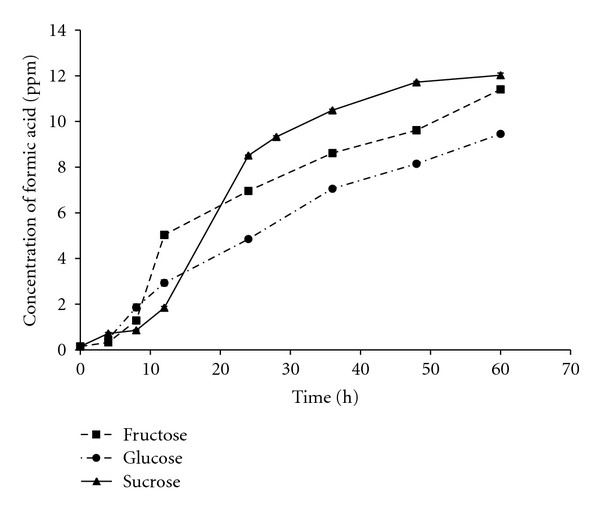
Variation in amount of organic acid produced with time on MSM supplemented with different carbon sources. The values represent average of three replicate measurements, and error bars represent standard deviation.

For the quantitative analysis of culture supernatant, different standard concentrations of formic acid were run, and the peak area corresponding to each concentration was calculated. The peak area was plotted against concentration in order to obtain a standard curve (Figure 3). This standard curve was used to calculate the amount of acid produced with time on minimal salt medium supplemented with different carbon sources. The culture supernatant of the bacterium was analyzed in a time-dependent manner and was again subjected to Reverse Phase HPLC analysis. It was found that the peak of the major product in bacterial culture supernatant increases with time. The retention time of the acid is similar to that of formic acid. The study of formic acid produced with different carbon sources as a function of time is shown in Figure 4. The highest amount of acid was produced in case of MSM supplemented with sucrose followed by fructose and glucose. 

## 4. Discussion 

The major carbon source in the effluent of beverage industry wastewater is sucrose [14]. Therefore, for the analysis of metabolic product produced during neutralization, a well-defined minimal salt medium containing sucrose and the two constituent monosaccharide sugars—glucose and fructose—were selected. The growth characteristics of strain 12/1 on minimal salt media supplemented with the three carbon sources show efficient neutralization concomitant with the growth (Figures 1(a) and 1(b)). The decrease in the pH of the growth medium is necessarily due either to the formation of acids or to the removal of bases [24]. 

The production of acids is well documented in case of bacteria grown on simple sugars. The metabolic products of some alkaliphilic members of genus* Bacillus *have been studied [15]. The major organic acid produced on sucrose carbon source in these studies was found to be acetic acid. The genome sequences of alkaliphilic bacillus species—*Bacillus pseudofirmus* OF4, *Bacillus halodurans*, and *Bacillus clausii*—also support this observation as all these species have a functional pyruvate to acetate conversion pathway. Formic acid is a common metabolite of neutrophilic bacteria in anaerobic conditions, while *B. circulans* var. *alkalophilus* produces up to 2 g/L of it even in aerobic cultures. Other volatile acids such as propionic, butyric, isobutyric and isovaleric acids are typical for strains *Bacillus alcalophilus ssp*. *halodurans*, *B. alcalophilus* and *Bacillus sp.* 17-1. Isobutyric and isovaleric acids have been reported in the media of several neutrophilic bacilli [16, 17]. But these acids, as well as propionic and butyric acids, are considered to originate from amino acids based on studies on *Clostridium sp*. [18]. Lactic and pyruvic acids are quite commonly produced by neutrophilic bacilli [16, 19, 20], but the production of succinic acid by *Bacillus* is rare [21]. Ethanol has not been detected in alkaliphilic bacilli [15] even though it is a typical product of glucose cultures of many neutrophilic bacilli [21, 22]. Thus alkaline growth conditions may affect the production of the neutral metabolites [15].

The initial studies on the metabolic products in case of *bacillus sp.* had been carried out using titrimetric procedure [15]. The increased buffering capacity of the bacterial culture supernatant around pH 5 which is the typical range of protonation of carboxylic acids was used to hypothesize that the medium contains carboxylic acids. In this study, we have used FT-IR spectroscopy to ascertain the functional group of compound(s) present in the culture supernatant. The FT-IR spectrograph showed the peaks characteristic of carbonyl group (at 1644.98 nm) and hydroxyl group (at 3436.74 nm) (Table 1) which suggests the presence of a chemical species consisting of hydroxyl and carbonyl group and is most likely to be carboxylic acid.

The Reverse Phase HPLC method was used to analyze the organic acids present in culture supernatant [23]. The HPLC conditions were chosen for best-reported resolution, that is, pH 2.2 and 1% acetonitrile. Reverse Phase HPLC method is advantageous because of the use of more inexpensive columns, easier manipulation of the analytical parameters to optimize the separation, and the analyses at room temperature [25]. The method was first used to calculate the retention time of acid standards chosen according to the literature survey. The order of elution of acids under these conditions was same as that reported in Tormo and Izco [23], but there was variation in the retention times observed in this study and that reported in Tormo and Izco [23]. This variation may be attributed to difference in HPLC conditions such as temperature 25–30°C in this study versus 24°C ± 1°C reported in Tormo and Iczo [23]. 

 The RP-HPLC of culture supernatant shows the presence of a single peak with absorbance at 211 nm which is characteristic absorption wavelength of organic acids. Thus the supernatant contains a single chemical species which is most likely organic acid. The comparison of retention time of this peak and the retention time of standard organic acids shows that the retention time is most similar to formic acid. This observation was further confirmed by spiking of formic acid in the culture supernatant which increases the peak area of the product (Figures 2(c) and 2(d)). The presence of formic acid in culture supernatant is in accordance with the metabolic products of some alkaliphilic members of genus* Bacillus *[15] as well as some saccharolytic anaerobic alkaliphilic bacteria such as *Halonatronum saccharophilum*, *Amphibacillus fermentum*, and *Amphibacillus tropicus* [26]. 

The maximum decrease in pH units occurring per unit time reported so far in alkaliphilic bacteria is 0.13 units per hour in case of *Bacillus circulans *var.* alkalophilus* [15] which is quite low as compared to more than two-unit decrease during initial 1 h of inoculation reported in this study. The large decrease of pH indicated a formation of acidic catabolism products. However, the rate of decrease of pH alone does not indicate the increase in concentration of acids [15]. Therefore quantitative analysis of the metabolic product of the bacterium was carried out using RP-HPLC. HPLC was preferred over GC as a comparison of GC and HPLC methods for determination of organic acids in culture supernatants of alkalophilic bacteria suggests that whereas the resolution of acids by GLC was excellent, the quantitative reproducibility by HPLC was better than GLC [27]. As expected, the concentration of acid produced was found to increase with the increase in incubation time (Figure 4). In fact, the amount of acid continued to increase much after the minimum pH was achieved. This is in accordance with the earlier studies of acid production in case of facultative and obligate alkaliphilic *Bacillus sp.* where acid production continued to increase even 30 h after the minimum pH was achieved [15]. The comparative analysis of the metabolic product produced in media supplemented with different carbon sources shows that although acid was present in same final concentration, the rate of acid production was highest in case of medium supplemented with sucrose followed by fructose and glucose (Figure 4). This is in accordance with the growth characteristics of the organism in the media supplemented with these sugars. 

## 5. Conclusion 

*Exiguobacterium sp.* strain 12/1 neutralizes the pH of the external medium by production of short chain organic acid-formic acid. Keeping in view the potential application of large-scale bioremediation of alkaline wastewaters, this alkali neutralization capacity of the bacterium for beverage industry wastewater can be considered as a first step towards exploitation of its commercialization potential. 

## Figures and Tables

**Table 1:** Result of FT-IR spectroscopy of the culture supernatant of strain 12/1.

Peak number	Peak type	Wave number (cm^−1^)	Inference
1	**Major**	**3436.74**	**Hydroxyl group**
2	Minor	2095.92	
3	**Major**	**1644.98**	**Carbonyl group**
4	Minor	1167.97	
5	Minor	1079.86	

**Table 2:** Retention time of standard organic acids. RT^a^ individual retention time, RT^b^ retention time in mixture. RT^c^ shows the retention times reported in Tormo and Izco [23].

S. no.	Organic acid	RT^a^	RT^b^	RT^a^-RT^b^	RT^c^
1	Formic acid	4.13	4.18	−0.05	2.56
2	Lactic acid	5.28	5.37	−0.09	3.57
3	Acetic acid	5.58	5.65	−0.07	3.76
4	Succinic acid	7.65	7.80	−0.13	5.68
5	Propionic acid	11.49	10.73	0.76	8.08
6	Isobutyric acid	23.40	23.35	0.05	—
